# Correlation between human ether‐a‐go‐go‐related gene channel inhibition and action potential prolongation

**DOI:** 10.1111/bph.13942

**Published:** 2017-08-11

**Authors:** P Saxena, M P Hortigon‐Vinagre, S Beyl, I Baburin, S Andranovits, S M Iqbal, A Costa, A P IJzerman, P Kügler, E Timin, G L Smith, S Hering

**Affiliations:** ^1^ Institute of Pharmacology and Toxicology University of Vienna Vienna Austria; ^2^ Institute of Cardiovascular and Medical Sciences University of Glasgow Glasgow UK; ^3^ Clyde Biosciences Ltd Glasgow UK; ^4^ Division of Medicinal Chemistry, Leiden Academic Centre for Drug Research Leiden University Leiden Netherlands; ^5^ Institute for Applied Mathematics and Statistics University of Hohenheim Stuttgart Germany; ^6^ Radon Institute for Computational and Applied Mathematics Austrian Academy of Sciences Vienna Austria

## Abstract

**Background and Purpose:**

Human ether‐a‐go‐go‐related gene (hERG; K_v_11.1) channel inhibition is a widely accepted predictor of cardiac arrhythmia. hERG channel inhibition alone is often insufficient to predict pro‐arrhythmic drug effects. This study used a library of dofetilide derivatives to investigate the relationship between standard measures of hERG current block in an expression system and changes in action potential duration (APD) in human‐induced pluripotent stem cell‐derived cardiomyocytes (hiPSC‐CMs). The interference from accompanying block of Ca_v_1.2 and Na_v_1.5 channels was investigated along with an in silico AP model.

**Experimental Approach:**

Drug‐induced changes in APD were assessed in hiPSC‐CMs using voltage‐sensitive dyes. The IC_50_ values for dofetilide and 13 derivatives on hERG current were estimated in an HEK293 expression system. The relative potency of each drug on APD was estimated by calculating the dose (D_150_) required to prolong the APD at 90% (APD_90_) repolarization by 50%.

**Key Results:**

The D_150_ in hiPSC‐CMs was linearly correlated with IC_50_ of hERG current. In silico simulations supported this finding. Three derivatives inhibited hERG without prolonging APD, and these compounds also inhibited Ca_v_1.2 and/or Na_v_1.5 in a channel state‐dependent manner. Adding Ca_v_1.2 and Na_v_1.2 block to the in silico model recapitulated the direction but not the extent of the APD change.

**Conclusions and Implications:**

Potency of hERG current inhibition correlates linearly with an index of APD in hiPSC‐CMs. The compounds that do not correlate have additional effects including concomitant block of Ca_v_1.2 and/or Na_v_1.5 channels. In silico simulations of hiPSC‐CMs APs confirm the principle of the multiple ion channel effects.

**Abbreviations**
APD: action potential duration
CiPA: comprehensive *in vitro* proarrhythmia assay
hERG: human ether‐a‐go‐go‐related gene
hiPSC‐CMs: human‐induced pluripotent stem cell‐derived cardiomyocytes
I_Kr_: delayed rectifier potassium current
TdP: torsade de pointes

## Introduction

The current paradigm of assessing drug‐induced pro‐arrhythmic risk is based on a link between drug‐induced human ether‐a‐go‐go‐related gene (hERG also known as K_v_11.1) channel blockade and QT‐interval prolongation; for review, see Sanguinetti and Tristani‐Firouzi (2006). The abnormal activity of cardiac myocytes such as early after‐depolarizations (EADs) is more likely to occur when the cardiac action potential (AP) is prolonged. EADs manifest as a single spike or oscillations of the membrane potential at the repolarising phase of the AP (Keating and Sanguinetti, 2001; Morita *et al.,* 2008; Liu *et al.,* 2012) and are commonly seen in patients with an acquired long‐QT syndrome (Veldkamp *et al.,* 2001; Pogwizd and Bers, 2004). EADs are pro‐arrhythmic because of their potential to induce dispersed refractory periods in cardiac tissue, which is a vital condition for the precipitation of arrhythmias. A link between EADs and *torsade de pointes* (TdP) has been previously studied (Volders *et al.,* 2000), and it is widely accepted that the prolongation of the QT interval is the precursor of EADs and TdP caused by many drugs (Hancox *et al.,* 2008; Sager *et al.,* 2014).

Many experimental and theoretical studies have been performed to investigate the ionic mechanisms of EADs in isolated cardiomyocytes (Zeng and Rudy, 1995; Sato *et al.,* 2010; Liu *et al.,* 2012). The repolarization phase of cardiac AP results from a complex interplay between several ionic currents such as inward sodium current (I_Na_), inward calcium current (I_CaL_) and several potassium currents mainly rapid delayed rectifier potassium current (I_kr_). EADs can be produced either by increasing the inward currents, mainly L‐type calcium current (I_CaL_), or reducing the outward currents (I_Kr_), or both. So, for example, a cell can be made susceptible to EADs by inhibiting I_Kr_ through hERG with dofetilide, activating the late sodium current (I_NaLate_) with veratridine or by increasing the conductance of I_CaL_ through Ca_v_1.2 channels with BAY K8644 (Horváth *et al.,* 2015). Drugs with unidirectional inhibition of inward and outward currents are generally unable to prolong AP duration (APD) and thus unlikely to induce EADs. Verapamil is one example that simultaneously inhibit I_Cal_ and hERG current without prolonging the QT interval (Zhang *et al.,* 1999).

Kramer J *et al.* (2013) have found that prediction of pro‐arrhythmogenity may be improved by considering the effect of drugs on currents from three key ion channels: hERG potassium channels (K_v_11.1), sodium channels (Na_v_1.5) and calcium channels (Ca_v_1.2). The development of multiple ion channel effect models leads to a significant reduction in false‐positive and false‐negative predictions when compared with hERG assays alone. Recently, the Cardiac Safety Research Consortium and the Food and Drug Administration proposed a new cardiac safety paradigm labelled as ‘comprehensive *in vitro* pro‐arrhythmia assay’ (CiPA). The new CiPA guidelines advocate studying the pharmacological effects of drugs on multiple ion channels that play an important role in shaping the ventricular AP (hERG, Na_v_1.5, Ca_v_1.2) instead of only hERG screening, and confirmation of electrophysiological effects using myocyte assays such as human‐induced pluripotent stem cell‐derived cardiomyocytes (hiPSC‐CMs).

Previous studies of pro‐arrhythmic effects of hERG inhibitors used a variety of chemical classes with different potencies and different selectivity. In this study, minor changes in the chemical structure of the highly potent and selective hERG inhibitor dofetilide generate compounds with a wide range of IC_50_ values. A remarkable linear relationship was observed between the IC_50_ value and the degree of AP duration change observed in hiPSC‐CM a relationship confirmed using an *in silico* model. The few derivatives not adhering to this linear relationship showed significant effects on Na_v_1.5 and Ca_v_1.2 ion channels.

## Methods

### Group sizes

Numbers (*n*) for all experiments are provided and refer to independent single measurements. Data subjected to statistical analysis have *n* of at least five per group. In the case of the APD, experiments on hiPSC‐CMs have a minimum of *n* = 4 in some cases. The *n* = 4 can discriminate a 15% change in APD_90_ (APD at 90% repolarization) with α = 0.95 and β = 0.2, from power calculations. The variability in APD values on a well‐to‐well basis (in 96‐well plate) was measured and can be expressed in terms of a coefficient of variation for CDI cells [commercially available from Cellular Dynamics International (CDI), Madison, WI, USA] after rate correction (1 Hz) is 0.08.

### Randomization

Randomization was not applicable, hence not performed.

### Blinding

Blinding of experiments is not applicable.

### Human‐induced pluripotent stem cell‐derived cardiomyocytes cell culture

Cryopreserved iCell hiPSC‐CMs (CDI, Lot no 1093711) were plated using iCell‐Plating Media (CDI, CMM‐100‐110‐001) by following the manufacturer's instructions. The cells were seeded at 25 000 cells per well in 96‐well glass‐bottomed plates (MatTek, p96G‐1.5‐5‐F) pre‐coated with 1:100 fibronectin (Sigma, F1141) in DPBS (Gibco, ThermoFisher Scientific, UK, 14 040–133) for 3 h at 37°C before cell plating. The plates were then incubated at 37°C, 5% CO_2_. Forty‐eight hours post‐thaw, 100% of the plating medium was replaced with CDI Maintenance Medium (CDI, CMC‐100‐120‐001), and further, 100% media changes were performed every 2 days after that. Optical recordings were performed 10–14 days post‐thaw at 37°C (Hortigon‐Vinagre *et al.,* 2016).

### Optical measurement of transmembrane potential signals using voltage‐sensitive dyes

Two hours before the experiments, the cells were transiently loaded with the voltage‐sensitive dye (VSD) di‐4‐ANEPPS (6 μM, 1 min at room temperature) in serum‐free media (DMEM, Gibco 11 966, supplemented with galactose 10 mM and sodium pyruvate 1 mM). Afterwards, the medium containing VSD was replaced by fresh serum‐free medium, and the cells were returned to the incubator. The multi‐well plate was placed in an environmentally controlled stage incubator (37°C, 5% CO2, water‐saturated air atmosphere) (Okolab Inc, Burlingame, CA, USA) of the CelIOPTIQ platform (Clyde Biosciences Ltd, Glasgow, Scotland). The di‐4‐ANEPPS fluorescence signal was recorded from a 0.2 × 0.2 mm area using a 40× (NA 0.6) objective lens. Excitation wavelength was 470 ± 10 nm using a light‐emitting diode (LED), and emitted light was collected by two photomultipliers (PMTs) at 510–560 and 590–650 nm respectively. LED, PMT, associated power supplies and amplifiers were supplied by Cairn Research Ltd (Kent, UK). The two channels of fluorescence signals were digitized at 10 kHz, and the ratio of florescence (short wavelength/long wavelength) was used to assess the time course of the transmembrane potential independent of cell movement (Knisley *et al.,* 2001). Baseline spontaneous electrical activity was recorded by capturing a 20 s segment of fluorescent signal prior to compound (drug) addition. Acute effects of dofetilide and derivatives were assessed by exposure to increasing drug concentration with matched vehicle controls for each concentration. A 20 s recording was then taken 30 min after exposure to the drug or vehicle with only one concentration applied per well. The records were subsequently analysed offline using proprietary software (CellOPTIQ). The procedure was repeated from four to five times, and parallel matched control (vehicle) measurements were taken on cardiomyocytes with equivalent concentrations of vehicle (DMSO). AP parameters were measured, including APD at 50, 75 and 90% repolarization (APD_50_, APD_75_ and APD_90_ respectively). Data are given as % change from control for the treated groups (vehicle, control and drug). This allowed a single comparison to be made at each concentration, and every experiment was performed with its own set of controls (vehicle). No data were used more than once.

### Cell culture and transient transfection tsA‐201 cells

HEK tsA‐201 cells were grown at 5% CO_2_ and 37°C to 80% confluence in Dulbecco's modified Eagle's/F‐12 medium supplemented with 10% (v·v^−1^) FCS and 100 U·mL^−1^ penicillin/streptomycin. Cells were split with trypsin/EDTA and plated on 35 mm Petri dishes (Falcon) at 30–50% confluence ~16 h before transfection.

### Patch‐clamp studies on hERG, Na_v_1.5 and Ca_v_1.2 channels

Currents through hERG channels (Anaxon GmbH) and Na_v_1.5 channels stably expressed in HEK293 cells were studied within 8 h of harvest in the whole‐cell configuration of the planar patch clamp technique (NPC‐16 Patchliner, Nanion Technologies GmbH, Munich, Germany), using an EPC 10 patch‐clamp amplifier (HEKA Elektronik Dr. Schulze GmbH, Lambrecht/Pfalz, Germany) (Milligan *et al.,* 2009). Currents were low‐pass filtered at 10 kHz using the internal Bessel filter and sampled at 25 kHz. The extracellular solution for hERG current recording contained 140 mM NaCl, 4 mM KCl, 2 mM CaCl_2_, 1 mM MgCl_2_,5 mM D‐glucose and 10 mM HEPES (pH 7.4) (Sigma‐Aldrich). The intracellular solution for hERG current recording contained 50 mM KCl, 10 mM NaCl, 60 mM KF, 20 mM EGTA and 10 mM HEPES (pH 7.2). The extracellular solution for measuring sodium currents in HEK cells stably expressing the human clone of Na_v_1.5 (GenBank M77235) contained 4 mM KCl, 20 mM NaCl, 1.8 mM CaCl_2_, 0.75 mM MgCl_2_, 5 mM HEPES, 120 mM choline chloride and pH 7.4 using NaOH. The intracellular solution for sodium current recording contained 120 mM CsF, 20 mM CsCl, 5 mM EGTA, 5 mM HEPES and pH 7.4 using CsOH. All chemicals were obtained from Sigma‐Aldrich Chemie GmbH (Taufkirchen, Germany). The compound solutions were applied by means of the automated NPC‐16 Patchliner planar patch‐clamp platform. Data acquisition was done using the PatchMaster software version 2.65 (HEKA Elektronik Dr. Schulze GmbH).

For barium current (I_Ba_) measurements through voltage‐gated Ca^2+^ channels, HEK tsA‐201 cells were co‐transfected with cDNAs encoding the rabbit Ca_V_1.2 α_1_‐subunit (GenBank X15539) with auxiliary β_2a_ (Perez‐Reyes *et al.,* 1992) as well as α_2_‐δ_1_ (Ellis *et al.,* 1988) subunits and GFP to identify transfected cells (see Beyl *et al.*, 2012, for details). The transfection of tsA‐201 cells was performed using the FUGENE6 Transfection Reagent (Roche Diagonstics GmbH, Mannheim, Germany) following standard protocols. The tsA‐201 cells were used until passage number 15. No variation in channel gating related to different cell passage numbers was observed. I_Ba_ were studied by manual patch‐clamping (Hamill *et al.,* 1981) using an Axopatch 200A patch clamp amplifier (Axon Instruments, Foster City) 36–48 h after transfection. The extracellular bath solution (in mM: BaCl_2_ 20, MgCl_2_ 1, HEPES 10, choline‐Cl 90) was titrated to pH 7.4 with methanesulfonic acid. Patch pipettes with resistances of 1 to 4 MΩ were made from borosilicate glass (Clark Electromedical Instruments, UK) and filled with pipette solution (in mM: CsCl 145, MgCl_2_ 3, HEPES 10, EGTA 10), titrated to pH 7.25 with CsOH. The drugs were applied to cells under voltage clamp using a microminifold perfusion system. Ca^2+^ channel block was estimated as peak I_*Ba*_ inhibition during a train of short (50 ms) test pulses from −80 mV at a frequency of 0.2 Hz. Patch clamp experiments to study hERG, Na_v_1.5 and Ca_v_1.2 currents were performed at room temperature (22–25°C). All data were digitized and saved to disc. Current traces were filtered at 5 kHz and sampled at 10 kHz. The pClamp software package (Version 7.0 Axon Instruments, Inc.) was used for data acquisition and preliminary analysis. Microcal Origin 7.0 was used for analysis, and sigmoidal curves were fitted using the Hill equation.

### In silico studies of hiPSC‐CMs’ action potentials

The cellular AP model of Paci *et al.* (2012) for ventricular hiPSC‐CMs was used for comparative computational studies of APD_90_ prolongation caused by dofetilide and its derivatives. These effects were described by the common pore block model in which the currents through the channels potentially sensitive to drugs were calculated with a coefficient equal to a fraction of channels free of drug:

$$k=\frac{1}{1+\frac{\left[D\right]}{{\mathrm{IC}}_{50}}}$$

All computations were performed in MATLAB R2015b. AP simulations were performed for a temperature of 310 K (37°C).

### Data processing and normalization

Origin 7.0 (Origin Lab Corp., Northampton, MA, USA) was employed for data analysis and curve fitting. The cumulative concentration–inhibition curves were fitted using the Hill equation:

$$\frac{I_{\text{Drug}}}{I_{\text{control}}}=\frac{1-A}{1+{\left(\frac{C}{{\mathrm{IC}}_{50}}\right)}^{\mathrm{nH}}}+A$$

where IC_50_ is the concentration at which hERG inhibition is half‐maximal; C is the applied drug concentration; A is the fraction of hERG current that is not blocked; and n_H_ is the Hill coefficient (Windisch *et al.,* 2011). Data are presented as mean ± SEM for at least five cells from two different batches or for three independent measurements with HEK293 cells.

### Statistical comparison

Statistically significant differences were calculated using Student's *t*‐tests and one‐way ANOVA and data from independent recordings. Only *P*‐values <0.05 were accepted as statistically significant. Linear correlation was used to confirm a linear relationship between hERG IC_50_ and APD data. The data and statistical analysis comply with the recommendations on experimental design and analysis in pharmacology (Curtis *et al*., 2015).

### Drugs

Dofetilide was obtained from Sigma, and its derivatives were prepared as previously described (Shagufta *et al.,* 2009). All derivatives were dissolved in DMSO to prepare a 10 mM stock and stored at −20°C. Drug stocks were diluted to the required concentration in extracellular solution on the day of each experiment. The maximal DMSO concentration in the bath (1%) did not affect Ca_v_1.2 or Na_v_1.5 currents in any of the preparations. (Supporting Information Fig. S1).

### Nomenclature of targets and ligands

Key protein targets and ligands in this article are hyperlinked to corresponding entries in http://www.guidetopharmacology.org, the common portal for data from the IUPHAR/BPS Guide to PHARMACOLOGY (Southan *et al.,* 2016), and are permanently archived in the Concise Guide to PHARMACOLOGY 2015/16 (Alexander *et al.,* 2015).

## Results

### Dofetilide derivatives library

**Figure 1:**
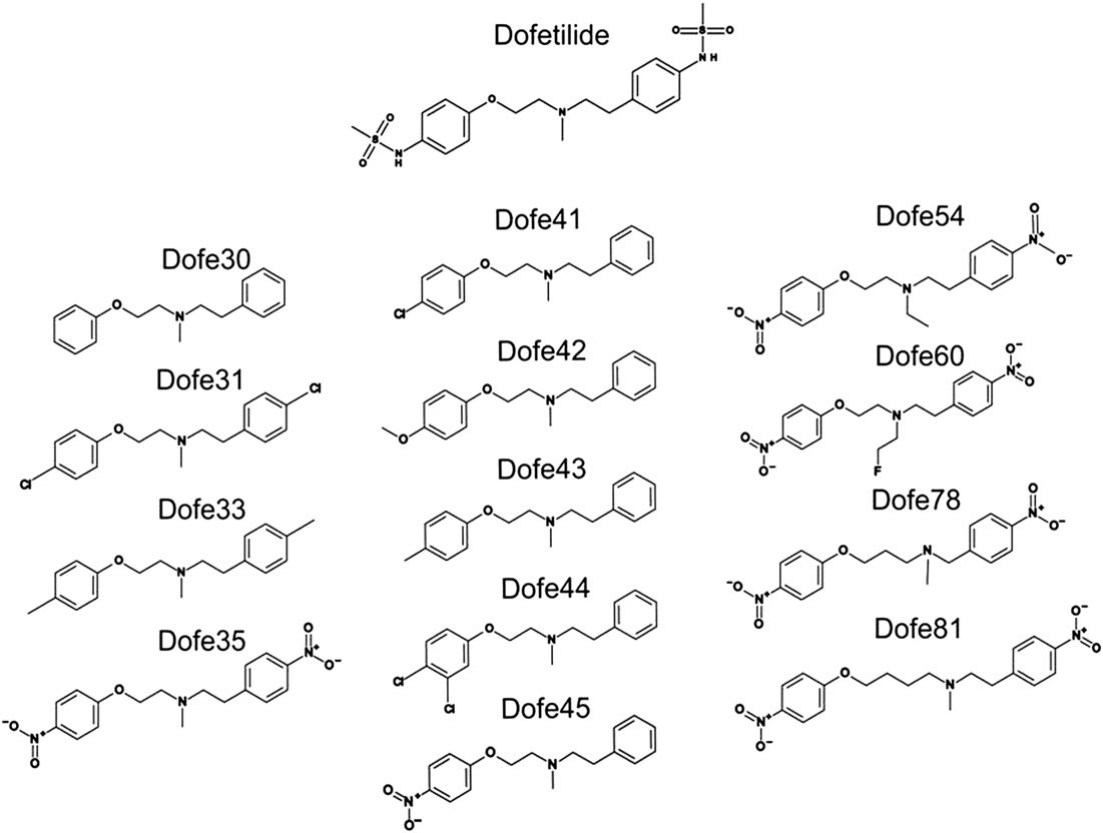
Chemical structures of dofetilide and its derivatives.

The small library of derivatives used in this study was previously described by Shagufta *et al.* (2009). The chemical structures of dofetilide and its 13 derivatives are shown in Figure 1. The structural modifications conserved the phenyl rings on both sides of the molecules and comprised the following: (i) attaching different substituents to the rings (all excluding Dofe30); (ii) changing the substituents on the protonated nitrogen (Dofe54, Dofe60); and (iii) varying chain length (Dofe78, Dofe81).

### Drug‐induced prolongation of APs in hiPSC‐CMs

**Figure 2:**
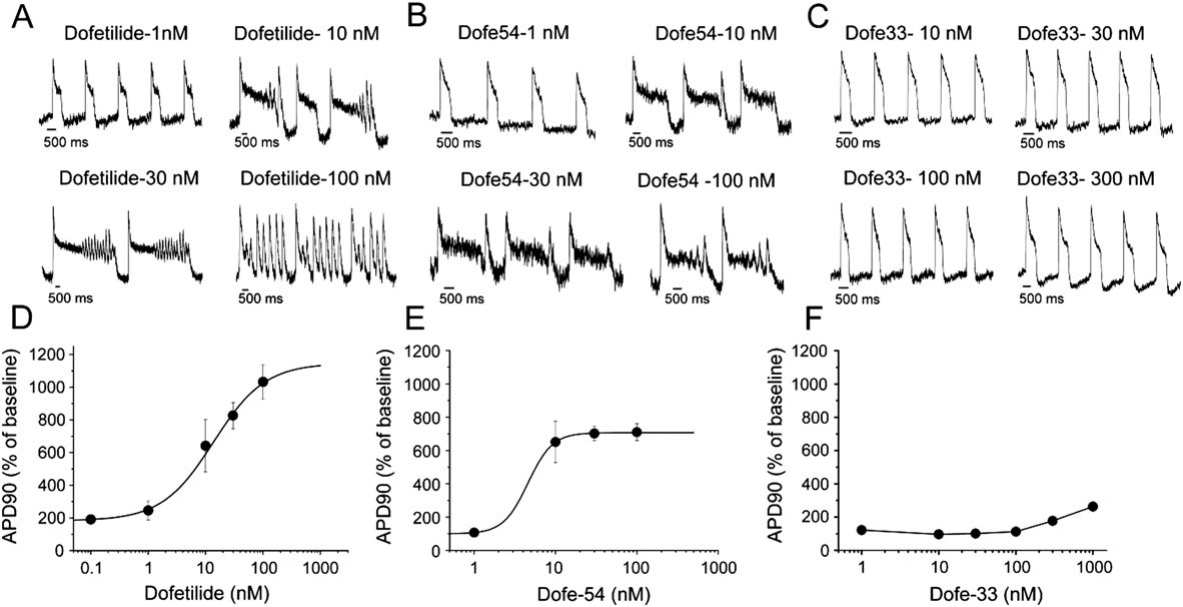
Effects of dofetilide and its derivatives Dofe54 and Dofe33 on AP characteristics in hiPSC‐CMs. Representative AP recordings of hiPSC cardiomyocytes after incubating with dofetilide, *n* = 4–5 (A), the high affinity derivative Dofe54, *n* = 4 (B) and the low affinity derivative Dofe33, *n* = 4 (C) and plots of APD_90_ as % of control versus concentrations of dofetilide, *n* = 4–5 (D), Dofe54, *n* = 4 (E) and Dofe33, *n* = 4 (F). The data points represent the mean ± SEM (see Table 1) and were fitted by a Hill equation for dofetilide and Dofe54. The data points for Dofe33 were connected by lines.

Effects of different concentrations of dofetilide and 13 derivatives on AP parameters were studied in hiPSC‐CMs. The changes in APD (as % of control) are given in Table 1. Figure 2 shows representative effects of dofetilide and two of its derivatives on spontaneous APs in cardiomyocytes. The derivative Dofe54 represents a potent pro‐arrhythmic compound and Dofe33 is an example with weak (if any) pro‐arrhythmic activity. Dofetilide‐induced concentration‐dependent lengthening of the AP was accompanied by incidence of EADs at concentrations of 10, 30 and 100 nM. The highest concentration used (100 nM) dramatically increased the spontaneous rate of myocyte contraction (Figure 2A).

**Table 1:** Changes in APD_90_ in hiPSC‐CMs after application of dofetilide and derivatives

Compound	0.1 nM	1 nM	10 nM	30 nM	100 nM	300 nM	1000 nM
Dofetilide	191(*n* = 6)	246(*n* = 4)	641(*n* = 4)	827(*n* = 4)	1032(*n* = 5)	–	–
Dofe54	–	107(*n* = 4)	651(*n* = 4)	702(*n* = 4)	710(*n* = 4)	–	–
Dofe81	–	117(*n* = 4)	389(*n* = 4)	858(*n* = 4)	1048(*n* = 5)	–	–
Dofe60	–	95(*n* = 4)	112(*n* = 4)	185(*n* = 6)	771(*n* = 4)	413(*n* = 6)	–
Dofe35	–	102(*n* = 4)	159(*n* = 4)	770(*n* = 4)	746(*n* = 4)	650(*n* = 5)	–
Dofe78	–	–	116(*n* = 4)	–	420(*n* = 4)	300(*n* = 4)	417(*n* = 4)
Dofe45	–	–	71(*n* = 4)	–	89(*n* = 4)	95(*n* = 4)	215(*n* = 4)
Dofe33	–	122(*n* = 4)	96(*n* = 4)	101(*n* = 4)	112(*n* = 4)	177(*n* = 4)	263(*n* = 4)
Dofe31	–	127(*n* = 4)	100(*n* = 4)	103(*n* = 4)	108(*n* = 4)	214(*n* = 4)	296(*n* = 4)
Dofe30	–	–	99(*n* = 4)	92(*n* = 4)	105(*n* = 4)	190(*n* = 4)	140(*n* = 4)
Dofe41	–	–	140(*n* = 6)	–	144(*n* = 5)	162(*n* = 5)	262(*n* = 4)
Dofe42	–	–	113(*n* = 4)	–	92(*n* = 4)	95(*n* = 4)	98(*n* = 4)
Dofe43	–	–	167(*n* = 5)	–	156(*n* = 5)	175(*n* = 5)	390(*n* = 4)
Dofe44	–	–	90(*n* = 4)	–	83(*n* = 4)	87(*n* = 4)	92(*n* = 4)

The values are presented as a % of control.

The potent derivative Dofe54 produced a slightly different pattern of AP distortion: smaller amplitude of oscillation during EADs and prolongation of APs at relatively low concentrations was observed. The 10 nM concentration induced approximately 700% prolongation of the AP (Figure 2B). The Dofe33 exhibited a negligible effect on APD_90_ prolongation until 100 nM. At a concentration of 300 nM, the APD_90_ was increased by approximately 170% of control. The maximal AP prolongation of 250% was observed at micromolar concentrations (Figure 2C). The concentration dependence of APD_90_ (in % to control) for dofetilide, Dofe54 and Dofe33 are shown in Figure 2D–F. The sigmoidal curves (Figure 2D, E) were fitted to the Hill equation.

**Figure 3:**
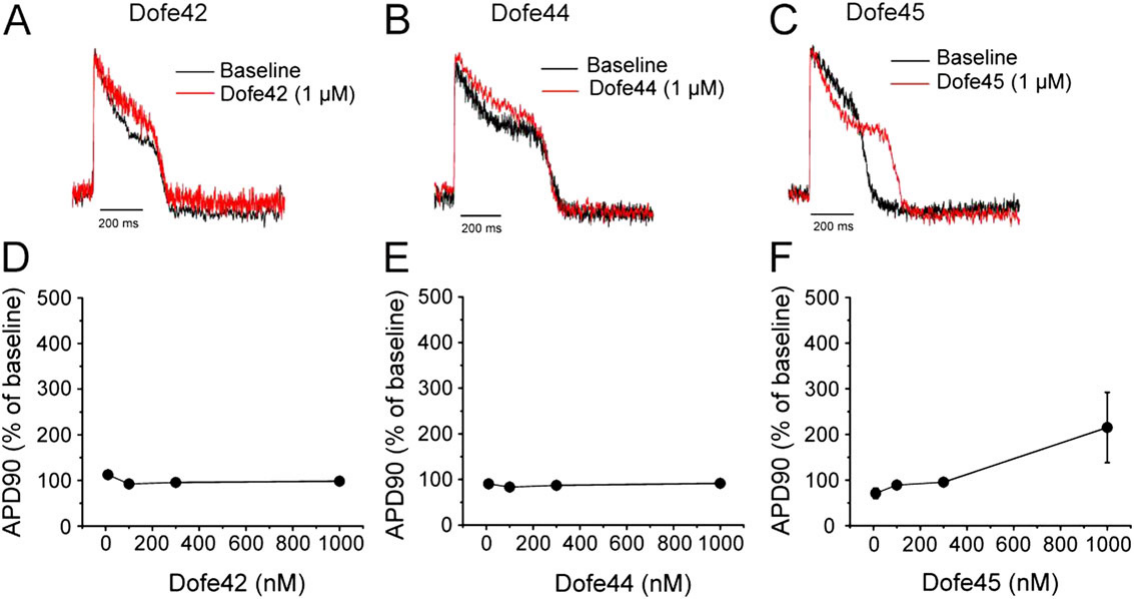
Effect of Dofe42, Dofe44 and Dofe45 on AP. (A–C) Representative AP traces of controls and in the presence of the indicated drugs. (D–F) Show dependence of APD_90_ on the concentration of indicated derivatives (*n* = 4–6, see Table 1).

Derivatives Dofe54, Dofe81, Dofe35, Dofe60 and Dofe78 had the most potent effects on APD (maximal level up to approximately 1000%), with incidence of EADs at the higher concentrations. In contrast, derivatives Dofe30, Dofe31, Dofe33, Dofe43, Dofe41 and Dofe45 exhibited relatively less effect on the APD_90_ without (if any) incidence of EADs. Derivatives Dofe42 (Figure 3A, D) and Dofe44 (Figure 3B, E) did not affect APD_90_ even at 1 μM while Dofe45 (Figure 3C, F) only slightly prolonged the AP.

### hERG channel inhibition by dofetilide and its derivatives

**Figure 4:**
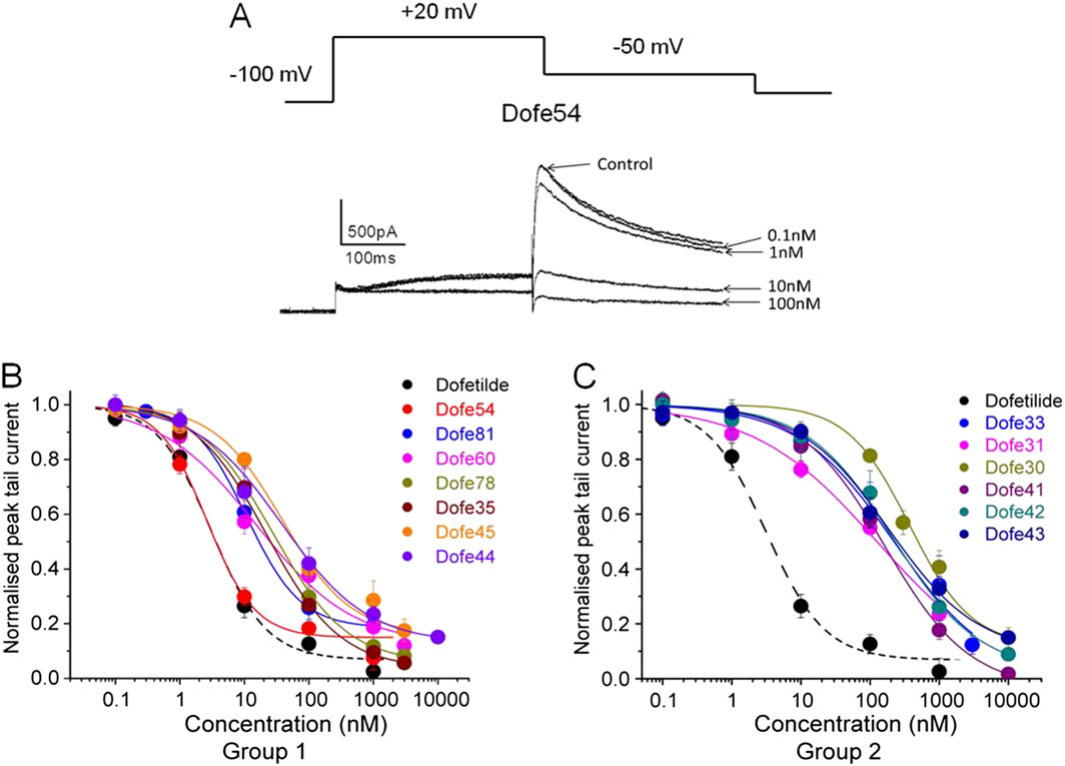
Effect of dofetilide and derivatives on potassium currents mediated through hERG channels expressed in HEK293 cells. (A) Representative current traces of control current (in the absence of drug) and in the presence of Dofe54 after steady state was reached at each concentration applied. The voltage protocol illustrated was applied every 3 s (A, upper panel). (B) Concentration‐inhibition curves for dofetilide (*n* = 5) and high affinity derivatives: Dofe54 (*n* = 5), Dofe81 (*n* = 6), Dofe60 (*n* = 5), Dofe78 (*n* = 7), Dofe35 (*n* = 8), Dofe45 (*n* = 5) and Dofe44 (*n* = 6). (C) Concentration‐inhibition curves for dofetilide and low affinity derivatives: Dofe33 (*n* = 6), Dofe31 (*n* = 7), Dofe30 (*n* = 7), Dofe41 (*n* = 8), Dofe42 (*n* = 8) and Dofe43 (*n* = 5). Peak tail current values (mean ± SEM, see Table 2) were fitted by the Hill equation.

hERG channel inhibition by dofetilide and derivatives was studied in HEK293 cell lines stably expressing hERG channels using an automated planar patch system (see Methods). After application of a given drug concentration, 0.3 Hz pulse trains were applied until a steady‐state of hERG current inhibition occurred. hERG current inhibition by Dofe54 is illustrated in Figure 4A. The concentration–inhibition relationships were analysed by plotting the normalized values of peak tail current versus peak tail steady current in the presence of the respective cumulatively applied compound concentrations (Figure 4B, C). Data points were fitted using Hill equation.

Figure 4 illustrates that dofetilide derivatives can be subdivided into the following: (i) high affinity derivatives hERG current inhibition with IC_50_ values ranging between 3 and 40 nM (Figure 4B); and (ii) low affinity derivatives with an IC_50_s of >100 nM (Figure 4C). The concentration–inhibition curves of group 1 derivatives were close to the dofetilide curve while curves of group 2 derivatives indicated reduced potency (approximately 10‐fold) of channel inhibition.

### Prolongation of AP correlates with potency to block hERG

The potency of dofetilide derivatives to prolong AP was related to their apparent affinity for hERG potassium channels. The drugs inhibiting hERG at lower concentrations prolonged the AP and induced EADs at lower concentrations (Table 2). In a first attempt, we failed, however, to observe a quantitative correlation between IC_50_ of hERG inhibition and the concentration that increased APD (D_150_) by 50%. Dofe42 and Dofe44 did not induce prolongation of AP and Dofe45 slightly prolonged the AP at high concentrations.

**Table 2:** Dofetilide and its derivatives: affinity for hERG potassium channels and concentration (D_150_) that prolongs the AP by 50%

Compound	MW	hERG IC_50_ (nM)	D_150_ (nM)
Dofetilide	441.567	3.1 ± 0.6 (*n* = 5)	0.04
Dofe54	395.84	2.6 ± 0.4 (*n* = 5)	4.3
Dofe81	409.87	10.7 ± 1.4 (*n* = 6)	2.5
Dofe60	413.83	15.3 ± 8.4 (*n* = 5)	21.3
Dofe78	345.35	28.2 ± 4.9 (*n* = 7)	20
Dofe35	381.82	22.1 ± 5.5 (*n* = 8)	8.3
Dofe45	336.82	38.6 ± 9.2 (*n* = 5)	538
Dofe33	319.88	221.6 ± 40.8 (*n* = 6)	215
Dofe31	360.71	125.2 ± 19.4 (*n* = 7)	151
Dofe30	291.82	296.9 ± 77.5 (*n* = 7)	213
Dofe41	326.26	164.6 ± 31.8 (*n* = 8)	157.2
Dofe42	321.84	213 ± 83.5 (*n* = 8)	650
Dofe43	305.84	184.3 ± 66.9 (*n* = 5)	99.3
Dofe44	360.71	38.1 ± 12.6(*n* = 6)	650

**Figure 5:**
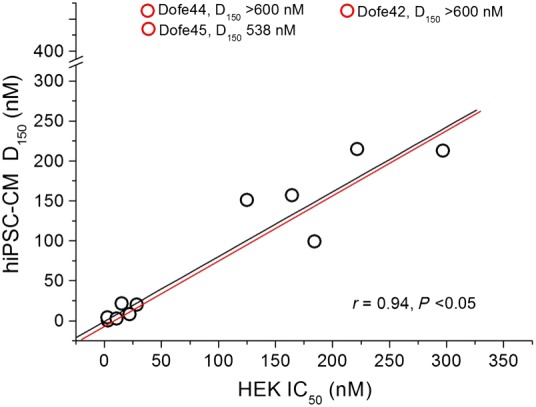
Correlation between D_150_ (concentration that prolongs AP in hiPSC‐CM by 50%) and IC_50_ (half‐maximal concentration inhibiting hERG channels in HEK293 cells). A significant linear correlation (*r* = 0.94, *P* < 0.05) was observed for 12 data points (black circles) including dofetilide and 11 derivatives. Derivatives Dofe45, Dofe44 and Dofe42 (red circles) were not included in the correlation analysis. Dofe45 prolonged the AP in hiPSC‐CM by 50% only at 538 nM and Dofe42 and 44 at >600 nM. The red line represents a prediction of the mathematical simulation of the hiPSC‐CM's AP (see Figure 8).

A plot of D_150_ versus drug affinities (IC_50_) is shown in Figure 5 (see also Table 2). Data points corresponding to derivatives that were not efficient at prolonging the AP (Dofe42, Dore44 and Dofe45; Figure 3) are illustrated as red circles in Figure 5. Excluding these data points from analysis led to a strong correlation (*r* = 0.94, *P* < 0.05) (Figure 5, black line) while taking them into account made the correlation non‐significant. The predicted relationship between IC_50_ and D_150_ by mathematical AP model is indicated by the red line. This model will be discussed in more detail later. In order to examine the possibility that additional block of inward currents may have counterbalanced hERG inhibition, we investigated effects of Dofe42, Dofe44 and Dofe45 on calcium (Ca_v_1.2) and sodium (Na_v_1.5) channels.

### Inhibition of Ca_v_1.2 by dofetilide and derivatives

**Figure 6:**
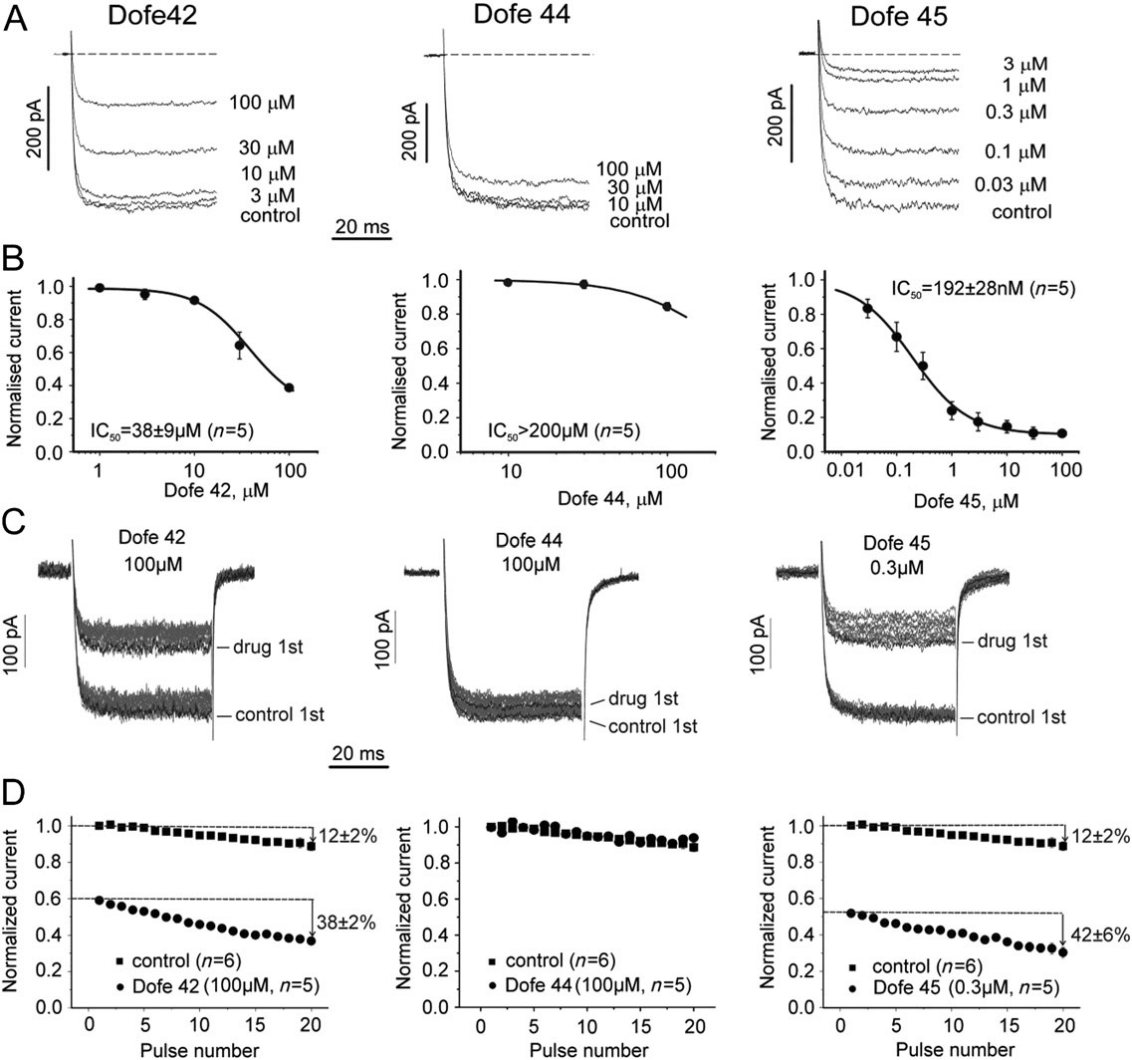
Inhibition of Ca_v_1.2 channel by dofetilide derivatives. (A) Superimposed barium currents through rabbit Ca_v_1.2 in control (black) and in the presence of indicated concentrations of Dofe42 (left), Dofe44 (middle) and Dofe45 (right). Barium currents were recorded in response to 50 ms pulses (0.2 Hz) from the holding potential of −80 to +10 mV. (B) Concentration‐dependence of peak I_Ba_ inhibition by Dofe42 (IC_50_ = 38 ± 9.3 μM, *n* = 5, left), Dofe44 (IC_50_ > 200 μM, *n* = 5, middle) and Dofe45 (IC_50_ = 192 ± 28 nM, *n* = 5, right). The IC_50_ values were obtained by fitting the data by the Hill equation. (C) Barium currents through Ca_v_1.2 during 1 Hz trains of 50 ms pulses from −80 to +10 mV under control conditions (absence of drug) and after 3 min incubation in the presence of the indicated concentrations of dofetilide derivatives. The first current in drug reflects the resting state inhibition. (D) Mean peak current amplitudes during 50 ms pulse trains in control and the presence of the indicated concentration of Dofe42, Dofe44 and Dofe45. The peak current decay after 20 pulses at 1 Hz in control indicates the development of inactivation. Peak current decay in the presence of Dofe42 (100 μM, 38 ± 2%, *n* = 5) and Dofe45 (100 nM, 42 ± 6%, *n* = 5) versus in control (12 ± 2%, *n* = 6) illustrates additional significant (*P* < 0.05) use‐dependent block.

Dofetilide itself does not inhibit Ca_v_1.2 even at a high concentration of 100 μM (Supporting Information Fig. S1a). Figure 6A illustrates the effects of Dofe42, Dofe44 and Dofe45 on Ca_v_1.2 at the indicated concentrations, and Figure 6B shows the corresponding concentration–inhibition curves obtained during continuous pulsing at 0.2 Hz. Dofe45 was identified as a potent Ca_v_1.2 blocker (IC_50_ = 190 ± 3 nM, Figure 6B, right panel) while Dofe42 and Dofe44 inhibited Ca_v_1.2 with comparably low potencies [IC_50_ of 38 ± 9.3 μM (Dofe42) and >100 μM (Dofe44)]. Use‐dependent channel inhibition was studied during trains of 1 Hz and 50 ms test pulses (from −80 to +10 mV). After the application of 20 test pulses in control (absence of drug), the cells were incubated for 3 min with drug at rest. Peak current inhibition during the first pulse (1st, Figure 6C) in the presence of the drug reflects ‘resting state’ block. Additional current inhibition during a subsequently applied pulse train illustrates use‐dependent block. Peak current inhibition in control and drug are compared in Figure 6D. Dofe42 and Dofe45 induced pronounced resting state block and additional use‐dependent block (compare last currents of the train in control and in drug). No use‐dependent block was observed for Dofe44 (Figure 6C, D middle panel).

### Inhibition of Na_v_1.5 by dofetilide derivatives

**Figure 7:**
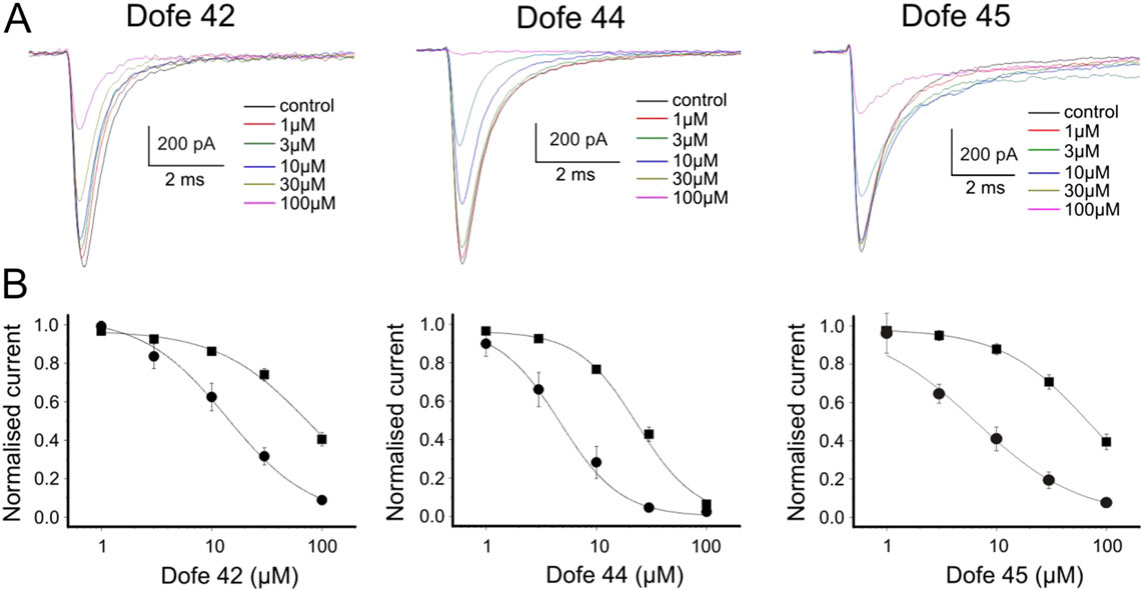
Inhibition of Na_v_1.5 by dofetilide derivatives. (A) Superimposed I_Na_ through human Na_v_1.5 in control (black) and in the presence of indicated concentrations of Dofe42 (left), Dofe44 (middle) or Dofe45 (right). Sodium currents were recorded in response to 20 ms pulses (0.2 Hz) from a holding potential of −140 to −10 mV. (B) Concentration‐dependence of peak I_Na_ inhibition at a holding potential of −140 mV (squares) and −80 mV (circles) yielding IC_50_ values for Dofe 42 of IC_50_ = 77.9 ± 9.7 (at −140 mV, *n* = 6) and IC_50_ = 13.8 ± 1.9 (at −80 mV, *n* = 5), Dofe 44 of IC_50_ = 23.3 ± 1.9 (at −140 mV, *n* = 6) and IC_50_ = 4.7 ± 2.0 (at −80 mV, *n* = 6) and Dofe 45 of IC_50_ = 69.7 ± 1.0 (at −140 mV, *n* = 6) and IC_50_ = 6.4 ± 1.0 μM (at −80 mV, *n* = 5).

Dofetilide (100 μM) did not inhibit Na_v_1.5 (Supporting Information Fig. S1b) while block was observed for Dofe42, Dofe44 and Dofe45. Figure 7A shows representative current traces illustrating the inhibition of Na_v_1.5 by derivatives at indicated concentrations. The concentration–inhibition curves for all three derivatives were first estimated at a holding potential of −140 mV where all Na_v_1.5 are available (Figure 7B, Wang *et al.,* 2015). Dofe44 inhibited cardiac sodium channels with an IC_50_ of 23.3 ± 1.9 μM (*n* = 6) compared with statistically less potent Dofe45 (IC_50_s of 69.7 ± 1.0 μM, *n* = 6, *P* < 0.05) and Dofe42 (77.9 ± 9.7 μM, *n* = 6, *P* < 0.05). However, the reported resting potentials of iPSC‐CM range between −75 and −63 mV (Hoekstra *et al.,* 2012) would induce substantial inactivation. In order to evaluate block of inactivated Na_v_1.5, we investigated I_Na_ inhibition at a holding potential of −80 mV where more than 60% of Na_v_1.5 were in an inactivated state (Wang *et al.,* 2015). Interestingly, the concentration–response curves where significantly shifted towards lower drug concentrations (Figure 7B, Dofe42: 5.6‐fold, Dofe44: fivefold and Dofe45: 7.7‐fold), suggesting that inactivated Na_v_1.5 are blocked with higher affinity. The application of test pulses at a higher frequency (1 Hz) did not induce additional channel inhibition.

### Computational studies support experimental findings

**Figure 8:**
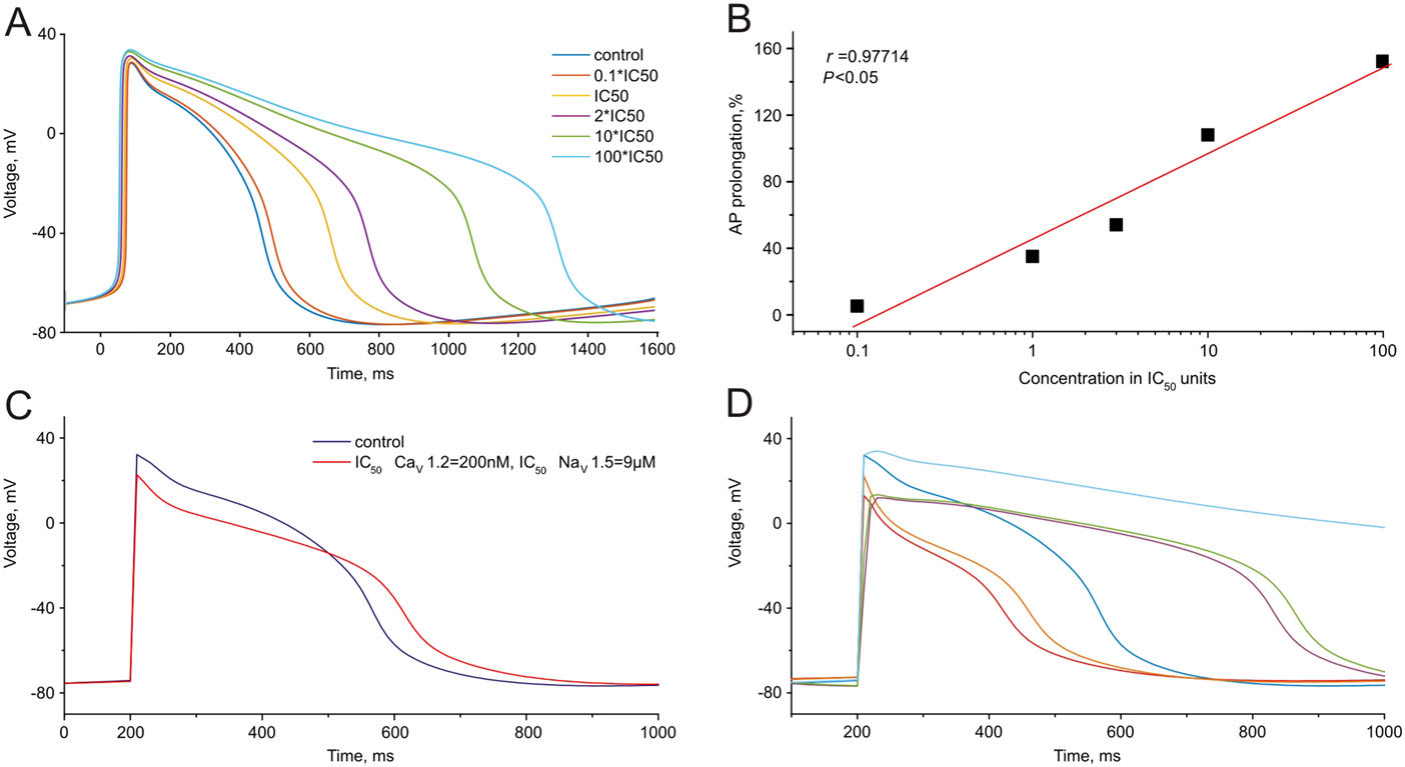
Simulation of hiPSC‐CM AP at indicated levels of hERG, Ca_v_1.2 and Na_v_1.5 channel inhibition. (A) Simulation of hiPSC‐CM APs for different levels of selective hERG channel inhibition. (B) Dependence of the calculated APD_90_ (as % of control) on the concentration of a selective hERG channel inhibitor. (C) Simulated APs at a Dofe45 concentration of 300 nM accounting for hERG inhibition (IC_50_ = 40 nM) and simultaneous inhibition of Ca_v_1.2 (IC_50_ = 200 nM) and Na_v_1.5 (IC_50_ = 8.9 μM). (D) Comparison of simulated APs at different IC_50_s of Ca_v_1.2 and Na_v_1.5 inhibition. Control AP is shown in dark blue and AP for selective hERG channel inhibition (IC_50_ = 40 nM) in light blue. Red 100 nM (Ca_v_1.2) and 1 μM (Na_v_1.5), orange 100 nM (Ca_v_1.2) and 10 μM (Na_v_1.5), magenta 500 nM (Ca_v_1.2) and 1 μM (Na_v_1.5), green 500 nM (Ca_v_1.2) and 10 μM (Na_v_1.5). See also Supporting Information Table S1 comparing the values used *in silico* AP models of adult ventricular myocytes and hiPSC‐CM.

The *in silico* AP model (Paci *et al.* 2012) for ventricular hiPSC‐CM was run with a pacing of 1 Hz until limit cycling was achieved in order to determine control APD_90_. In the first series of calculations, we have described a prolongation of AP under inhibition of hERG potassium channels. The drug dose D was set as a multiple of IC_50_, that is, D = x × IC_50_, enabling the use of the nonlinear forward mapping F: x → APD_90_(x). The factor x corresponding to a prolongation of the control APD_90_ by 50% was determined by solving (Engl *et al.*, 2009) the nonlinear inverse problem F(x) = 1.5 × (control APD_90_). The predicted relationship between IC_50_ and D_150_ is shown in Figure 5 (red line). Figure 8A displays AP simulations at different levels of inhibition of the hERG channel. The APD exhibited a linear correlation with logarithm of concentration of hERG channel blocker (Figure 8B).

In order to test whether inhibition of inward currents compensates for the APD changes seen with hERG channel block, we simulated APs for different concentrations of half‐maximal Ca_v_1.2 and Na_v_1.5 inhibition (for Ca_v_1.2, IC_50_ = 200 nM and Na_v_1.5, IC_50_ = 10 μM). Both IC_50_s are characteristic for Dofe 45 (Table 2, Figures 6 and 7). The simulation (Figure 8C) surprisingly coincides with experimental records (Figure 3C). The ‘selective inhibition’ of the hERG channels by Dofe45 would induce a substantial prolongation of the AP. Figure 8D illustrates the sensitivity of the APD to Ca_v_1.2 and Na_v_1.5 inhibition at different IC_50_s. Inhibition of either Ca_v_1.2 or Na_v_1.5 caused shortening of APD, the largest effects seen on Ca_v_1.2 inhibition (Figure 8D red and orange AP).

## Discussion and conclusion

Potential pro‐arrhythmic effects in early stages of drug development have often been assessed solely by examining hERG channel block. The principle role of hERG channel block for AP repolarization and its consequences have been extensively discussed (Sanguinetti and Tristani‐Firouzi, 2006). The new CiPA paradigm proposes that drugs should be tested by screening multiple ion channels including I_Kr_, I_Ks_ and I_K1_ as well as I_NaLate_ and I_CaL_ and predicting their effect on the human APD using *in silico* models to integrate the effects of a number of ion channels (Sager *et al.,* 2014). The CiPA scheme also suggests analysing pro‐arrhythmic effects using human iPSC‐derived cardiac muscle as a surrogate for human myocardium. However, not all ion channels expressed in human myocardium are equally well represented in hiPSC‐CM. In particular, studies suggest that I_K1_, I_Ks_ and I_NaLate_ currents have minimal contributions to the electrophysiology of the iPS cells (Paci *et al.*, 2012). The Na_v_1.5 channel that generates the upstroke phase and the Ca_v_ 1.2 responsible for maintaining the plateau phase of AP are known to be active. At the end of the plateau phase and beginning of repolarization, inward currents are small (largely inactivated) and countered by the activation of outward K^+^ currents, predominately hERG, which is responsible for initiating the repolarization phase, is well represented in iPS cell. We have previously reported the absence of I_NaLate_ effect in the presence of ranolazine in hiPSC‐CM (Hortigon‐Vinagre *et al.,* 2016), which shows that presence of I_NaLate_ in hiPSC is unlikely. Yang *et al.* (2014) reported an enhancement of I_Na‐L_ by dofetilide after chronic (5 h) drug exposure. Drug effects in our experiments were, however, studied a after short‐time (several minutes) of application and no increase in I_NaLate_ was observed. It is under discussion whether commercial hiPSC cell lines contain a range of cell types or simply broad‐spectrum features. The majority of hiPSC cells appear to have ventricular phenotype, and they are likely to operate as a functional syncytium *via* gap junction links (Bett *et al.,* 2013; Kane *et al.,* 2016).

Combining *in silico* studies with hERG (and other ion channels) inhibition and effects on APD should enable a more profound understanding of pro‐arrhythmic potential. To test this concept, we compared the prolongation of APs of hiPSC‐CM by the selective hERG inhibitor dofetilide and 13 derivatives with respect to their potencies to inhibit hERG (Figure 5). Derivatives retained the common scaffold of dofetilide while changing the functional group on both the ends or modifying the central nitrogen or altering the length of the molecule. The 13 derivatives inhibited hERG potassium channels with IC_50_s ranging from 3 to 300 nM (Figure 4B, C). Examining the effects of these derivatives on hiPSC‐derived cardiac myocyte APD revealed a correlation between the concentration (D_150_) inducing a 50% increase of APD_90_ of the cardiac AP with half‐maximal concentrations (IC_50_s) of hERG channel inhibition (Figure 5 and Supporting Information Fig. S2).

There was no correlation between the *K*_i_ values (affinity of derivatives to hERG estimated in radioligand studies; Shagufta *et al.,* 2009), and IC_50_s measured in electrophysiological experiments (see Supporting Information Table S2) was observed. All derivatives (except Dofe30) were similarly active in the binding study while IC_50_s measured in patch clamp experiments varied over two orders of magnitude (from 2.6 to 296 nM, Table 2). The lack of correlation between *K*_i_ and IC_50_ indicates that the interaction of these derivatives with their binding pocket is not the only determinant of hERG channel inhibition (Saxena *et al.,* 2016). The *K*_i_ value reflects the affinity of a derivative for the binding pocket putatively located in the channel pore while the IC_50_s estimated in patch‐clamp studies are affected by the following: (i) channel state‐dependent drug effects (Fernandez *et al.,* 2004; Sanguinetti and Tristani‐Firouzi, 2006; Stork *et al.,* 2007; Perry *et al.,* 2010; Windisch *et al.,* 2011); (ii) their ability to pass the entrance barrier or leave the channel cavity; and (iii) their affinity to the binding pocket within the channel. In this regard, it is interesting to note that the IC_50_s estimated from hERG inhibition in functional studies are in a reciprocal relation to the molecular weight of the tested derivatives (Supporting Information Fig. S3). It is tempting to speculate that the dependence of IC_50_ on the molecular size is caused by an energetic barrier at the channel pore entrance. In such a scenario, bulkier molecules with higher molecular weight leave the channel with lower probability, resulting in lower off rates and correspondingly in lower IC_50_ values.

Three derivatives (Dofe42, 44 and 45) failed, however, to fit a linear correlation (Figure 5). We hypothesize that the ineffectiveness of derivatives Dofe45, Dofe44 and Dofe42 to prolong the AP might be due to their interference with Ca_v_1.2, Na_v_1.5 and potentially other ion channels. Dofe45 was subsequently shown to be a potent inhibitor of Ca_v_1.2. In a first series of experiments, performed at a low stimulus frequency (0.2 Hz), this derivative inhibited Ca_v_1.2 with an IC_50_ of 190 ± 3 nM (Figure 6A, B). It is well established that open and inactivated channels may have a higher affinity for inhibitors than channels in the resting state (Hondeghem and Katzung, 1977). Therefore, additional measurements were made at a higher frequency of (1 Hz), which is comparable with the beating frequency of iPSC‐CM. The shorter (50 ms) pulses (1 Hz) revealed some additional use‐dependent channel inhibition by Dofe42 and 45 (Figure 6C, D). Thus, 1 Hz pulsing can enhance Cav1.2 inhibition due to additional block of open and/or inactivated channels. However, both derivatives inhibited Ca_v_1.2 predominantly in the resting state (Figure 6). A comparison of I_Na_ inhibition at −140 and −80 mV close to the resting potential of iPSC‐CM, where more than 60% of Na_v_1.5 channels are in an inactivated state (Hoekstra *et al.,* 2012; Wang *et al.,* 2015), revealed that Dofe42, Dofe44 and Dofe45 preferentially inhibit inactivated Na_v_1.5. This study is mainly focused on primary targets of these derivatives like I_Na_ and I_CaL_, and it is very unlikely that these derivatives would have an effect on secondary targets such as I_Ks_, Na/K pump, NCX and/or SR Ca^2+^ release.

As shown in Figures 5 and 8, our *in silico* studies on the AP model (Paci *et al.* 2012) at a resting potential of −80 mV reproduced the link between hERG inhibition (IC_50_) and prolongation of the AP (D_150_). Furthermore, accounting for inhibition of hERG, Cav1.2 and Nav1.5 by Dofe45 reproduced the principal features of AP changes observed on hiPSC‐CM (compare Figures 3C and 8C). The acceleration of early repolarization (phase 1) and inhibition of the AP overshoot are obviously caused by simultaneous inhibition of sodium channels while the prolongation of the AP was predominantly balanced by simultaneous block of Ca_v_1.2. Hence, as illustrated in Figure 8D, selective hERG inhibition by 300 nM Dofe45 would induce a more pronounced AP prolongation. The inability of Dofe42 and Dofe44 to prolong the AP is hard to explain exclusively by inhibition of Ca_v_1.2 and Na_v_1.5 as these channels appear to be blocked only at high concentrations. But the conditions of the ion channel assay are not the same as those of the iPSC‐CMs. The oscillatory voltage changes and the temperature will almost certainly alter the level of activation/inactivation of the currents. Both Na_v_1.5 and Ca_v_1.2 show voltage‐ and time‐dependant effects of drugs, and inhibition of inactivated Na_v_1.5 by Dofe42 and Dofe44 was stronger at −80 mV relative to −140 mV (Figure 7). Furthermore, Ca_v_1.2 showed use‐dependent block of by Dofe42 (Figure 6C, D). Therefore, the precise effect of drugs on both of these channels in the context of an AP in IPSC‐CMs is difficult to assess. Also, while Ca_v_1.2 and Na_v_1.5 are the most likely candidates for alternative drug actions, these derivatives may also modulate other ion channels that contribute to the AP shape.

The implications of this work are that potency of hERG current inhibition correlates linearly with an index of APD in hiPSC‐CMs. This simple relationship, confirmed *in silico*, allows data gained in one standard assay to predict the effect on another, that is, the IC_50_ of a drug in an ion channel hERG screen predicts the dose required to increased APD_90_ in iPSC‐CMs or *vice versa*. Furthermore, compounds that do not correlate will have additional effects including concomitant block of Ca_v_1.2 and/or Na_v_1.5 channels. Finally, the study shows that while *in silico* simulations can confirm the principle of the effects of Ca_v_1.2 and Na_v_1.5 inhibition on APD_90_, more comprehensive voltage clamp data are required to accurately predict the consequences of Ca_v_1.2 and Na_v_1.5 block on AP shape and duration *in silico*.

### Limitations

In the myocardium and hiPSC‐CM hERG, Ca_v_1.2 and Na_v_1.5 channels function under different conditions than in patch clamp experiments on mammalian cells. In order to relate patch clamp data to the hiPSC‐CM assay, it would be desirable to study these ionic currents at the beating frequency of hiPSC‐CM (~1 Hz) at 37°C. But most patch clamp ion channel assays place constraints on the design of the pulse protocol. As illustrated in Figure 6C, D, continuous 1 Hz pulsing with even short (50 ms) test pulses results in peak current decay of calcium currents caused by channel inactivation. Application of longer test pulses (e.g. 300 ms, corresponding to the length of the ventricular cardiac AP) at 1 Hz leads to inactivation by 30 and 40%, during a train of 20 pulses. Further optimization of experimental (temperature, test pulse length and shape, holding potential, pacing frequency, etc*.*) and theoretical conditions (analysis of participation of additional ionic currents in AP shaping) is required to achieve a higher level of congruence between the different assay data.

## Author contributions

P.S., M.P.V.H., A.C., S.B., I.B. and S.M.I. performed the experiments; P.S., M.P.V.H., A.C., S.B., A.P.I., P.K., E.T., G.L.S. and S.H. designed the study; P.S., M.P.V.H., A.C., S.B., A.P.I., P.K., E.T., G.L.S. and S.H. analysed data; P.S., M.P.V.H., E.T., P.K., G.L.S. and S.H. wrote the paper.

## Conflict of interest

The authors declare no conflicts of interest.

## Declaration of transparency and scientific rigour

This Declaration acknowledges that this paper adheres to the principles for transparent reporting and scientific rigour of preclinical research recommended by funding agencies, publishers and other organisations engaged with supporting research.

## Supporting information


**Figure S1** Dofetilide (100 μM) does not inhibit Cav1.2 or Nav1.5.
**Figure S2** Estimation of dose required to prolong the action potential by 150% (D_150_).
**Figure S3** Relationship between potencies of dofetilide derivatives to inhibit hERG (IC_50_) and their MW (MW).
**Table S1** Major maximal conductance of ion channels used for AP simulations of human embryonic stem cell‐derived myocytes described in Paci *et al.* (2012) and corresponding values used for adult ventricular cardiomyocyte models.
**Table S2** Potencies of dofetilide derivatives to inhibit hERG potassium channels estimated in patch clamp experiments and Ki values from binding studies (from Shagufta *et al.* 2009) in relation to MW.
