# Supplementary material for: Correlation between human ether‐a‐go‐go‐related gene channel inhibition and action potential prolongation
Source: Br J Pharmacol. 2017 Aug 11;174(18):3081–93. doi: 10.1111/bph.13942 (PMC5573420; doi:10.1111/bph.13942)
Supplement: Supplementary file 1 — Figure S1 Dofetilide (100 μM) does not inhibit Cav1.2 or Nav1.5. Figure S2 Estimation of dose required to prolong the action potential by 150% (D150). Figure S3 Relationship between potencies of dofetilide derivatives to inhibit hERG (IC50) and their MW (MW). Table S1 Major maximal conductance of ion channels used for AP simulations of human embryonic stem cell‐derived myocytes described in Paci et al. (2012) and corresponding values used for adult ventricular cardiomyocyte models. Table S2 Potencies of dofetilide derivatives to inhibit hERG potassium channels estimated in patch clamp experiments and Ki values from binding studies (from Shagufta et al. 2009) in relation to MW. [file BPH-174-3081-s001.docx]

**Supplemental Material**

**Figure S1 Dofetilide (100 µM) does not inhibit Cav1.2 or Nav1.5**

**
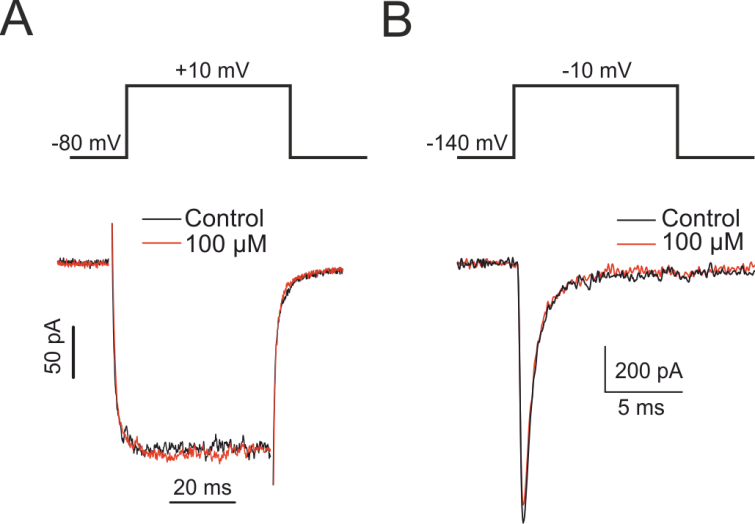
**

(A) Superimposed barium currents through rabbit Cav1.2 in control (black) and in the presence of 100 μM of dofetilide (red). (B) Superimposed I_Na_ through human Nav1.5 in control (black) and in presence of 100 μM dofetilide (red).

**Figure S2 Estimation of dose required to prolong the action potential by 150% (D_150_)**

**
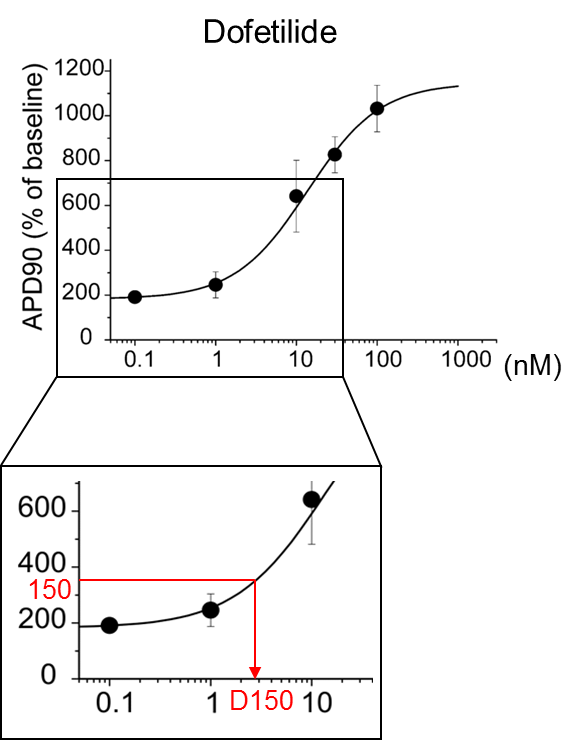
**

**Figure S3 Relationship between potencies of dofetilide derivatives to inhibit hERG (IC_50_) and their molecular weight (MW)**





**Table S1 Major maximal conductance of ion channels used for AP simulations of human embryonic stem cell-derived myocytes described in Paci et al (2012) and corresponding values used for adult ventricular cardiomyocyte models.**

| Currents | Late human embryonic stem cell-derived model | Adult ventricular cardiomyocyte models |
| --- | --- | --- |
| I_Na_ max conductance (S/F) | 14838 | 14838 |
| I_Ca_ permeability (dm3/(F⋅s)) | 0.0739 | 0.175 |
| I_Kr_ max conductance (S/F) | 134.4 | 96 |
| I_K1_ max conductance (S/F) | 1154.508 | 5405 |
| I_Ks_ max conductance (S/F) | 15.7 | 157 |

**Table S2 Potencies of dofetilide derivatives to inhibit hERG potassium channels estimated in patch clamp experiments and Ki values from binding studies (from Shagufta et al 2009) in relation to molecular weight**

| **Compound** | **MW** | **IC_50_ (nM), patch clamp** | **K_i_ (nM)  binding studies** |
| --- | --- | --- | --- |
| Dofetilide | 441.567 | 3.1 ± 0.6 (n=5) | 4.1± 0.9 |
| Dofe54 | 395.84 | 2.6 ± 0.4 (n=5) | 1.2 ± 0.6 |
| Dofe81 | 409.87 | 10.7 ± 1.4 (n=6) | 1.8 ± 1.6 |
| Dofe60 | 413.83 | 15.3 ± 8.4 (n=5) | 3.1 ± 1.0 |
| Dofe78 | 345.35 | 28.2 ± 4.9 (n=7) | 5.1 ± 1.9 |
| Dofe35 | 381.82 | 22.1 ± 5.5 (n=8) | 5.7 ± 3.0 |
| Dofe45 | 336.82 | 38.6 ± 9.2 (n=5) | 3.1 ± 1.9 |
| Dofe33 | 319.88 | 221.6 ± 40.8 (n=6) | 5.1 ± 4.7 |
| Dofe31 | 360.71 | 125.2 ± 19.4 (n=7) | 4.2 ± 1.6 |
| Dofe30 | 291.82 | 296.9 ± 77.5 (n=7) | >10,000 |
| Dofe41 | 326.26 | 164.6 ± 31.8 (n=8) | 3.1 ± 1.9 |
| Dofe42 | 321.84 | 213 ± 83.5 (n=8) | 3.3 ± 2.0 |
| Dofe43 | 305.84 | 184.3 ± 66.9 (n=5) | 7.9 ± 7.2 |
| Dofe44 | 360.71 | 38.1 ± 12.6 (n=6) | 2.4 ± 2.2 |
